# Construction and validation of a predictive model for the risk of prolonged preoperative waiting time in patients with intertrochanteric fractures

**DOI:** 10.3389/fmed.2024.1503719

**Published:** 2025-01-17

**Authors:** Rui Gong, Xi-min Jin, Lian-you Xu, Zhi-meng Zhang, Dao-tong Yuan, Wen-peng Xie, Yong-kui Zhang

**Affiliations:** ^1^First Clinical College, Shandong University of Traditional Chinese Medicine, Jinan, China; ^2^Department of Orthopedic Surgery, Afliated Hospital of Shandong University of Traditional Chinese Medicine, Jinan, Shandong, China

**Keywords:** intertrochanteric fractures, preoperative waiting time, risk factors, nomograms, model

## Abstract

**Background:**

Intertrochanteric fractures are one of the most common types of hip fractures, with delayed surgical treatment beyond 48 h associated with increased postoperative complications and mortality, especially in older adults. This study aimed to develop a predictive model for delayed preoperative waiting times in intertrochanteric fracture cases, based on previous research, to offer a valuable reference for clinical decision-making.

**Methods:**

A retrospective analysis was conducted on 1,116 patients with intertrochanteric fractures admitted to the Affiliated Hospital of Shandong University of Traditional Chinese Medicine for internal fixation surgery from January 2017 to January 2024. Patient demographic data and clinical examination results were collected. A logistic regression model was used to construct a predictive model, which was then visualized through a nomogram. The model’s performance was subsequently validated.

**Results:**

The predictive model developed from 728 patients in the training cohort, identified key predictors, including age, sex, lower extremity deep vein thrombosis, injury location, and biochemical markers. The model demonstrated strong discriminative ability, with an area under the receiver operating characteristic curve of 0.749 (95% confidence interval: 0.621–0.801) for the training set, and 0.745 in the validation set. Calibration curves indicated that the predicted risk of surgical delay closely aligned with observed outcomes. Furthermore, decision curve analysis verified the model’s clinical utility, demonstrating its effectiveness in guiding treatment decisions.

**Conclusion:**

The nomogram model developed in this study provides a reliable tool for predicting delayed surgical intervention in patients with intertrochanteric femur fractures. It offers clinicians a valuable reference to anticipate delays in surgical treatment and aids in the formulation of more timely and appropriate treatment strategies, potentially improving patient outcomes.

## Introduction

Intertrochanteric fractures of the femur are associated with high mortality rates and a significant incidence of postoperative complications. In older adults, these fractures are often referred to as “the last fracture of life” due to their severe impact on health and mobility. Globally, the number of hip fractures in individuals aged more than 50 years is projected to reach 4.5 million by 2025 (1). In China alone, with the rapid pace of economic and social development and an aging population, approximately 500,000 hip fractures occur annually (2). These fractures impose substantial individual medical costs and socioeconomic burdens. The relationship between preoperative waiting time and improved surgical outcomes, reduced postoperative complications, but a better prognosis remains controversial (3, 4). However, evidence suggests that reducing the preoperative waiting period leads to better patient outcomes (5). Reduced preoperative waiting time has been shown to be beneficial in reducing the incidence of postoperative complications in elderly hip fracture patients and in facilitating postoperative functional recovery (6, 7). Previous studies have demonstrated that comorbidities are responsible for prolonged preoperative waiting times (8). Despite this, no predictive models exist to anticipate the extension of preoperative waiting time in cases of intertrochanteric femur fractures. A model that predicts prolonged waiting times based on information collected at admission is crucial to help clinicians make informed decisions and develop more effective treatment strategies. Nomogram models have proven useful for predicting diagnoses and prognoses in various diseases. To our knowledge, no nomogram model has been developed to predict prolonged preoperative waiting times in patients with intertrochanteric femur fractures. Therefore, this study aimed create an accurate and reliable nomogram model to assist clinicians in predicting delays in surgical treatment and optimizing patient care.

## Methods

### Patients

In this study, we collected basic information, clinical data, and laboratory findings at the time of admission from 1,129 patients with intertrochanteric fractures who were hospitalized in the Department of Orthopedics at the Affiliated Hospital of Shandong University of Traditional Chinese Medicine. All cases were collected by searching the database within our hospital. These patients underwent surgical treatment from January 2017 to January 2024. The study was approved by the Ethics Committee of the Affiliated Hospital of Shandong University of Traditional Chinese Medicine. The inclusion criteria for the study were as follows: (1) age ≥ 18 years; (2) confirmed diagnosis of unilateral intertrochanteric femur fracture based on X-ray or CT examination; (3) hospitalization and subsequent surgical treatment with internal fixation; (4) length of stay <2 weeks. The exclusion criteria were as follows: (1) bilateral intertrochanteric femoral fractures; (2) multiple fractures; (3) pathologic fractures (4) undergoing two or more surgeries; (5) incomplete data. Exclusion criteria were developed based on our previous studies (9). And we excluded cases of selection bias from interfering with the outcome. A total of 13 patients were excluded based on these criteria, leaving 1,116 patients for inclusion in the study. Out of all enrolled patients, 70% were randomly allocated to the training set and the remaining 30% were assigned to the validation set. The data was split performed using stratification. The training set was utilized to develop the nomogram model, whereas the validation set was used to assess the model’s performance.

### Data collection

Two clinical orthopedic surgeons reviewed all cases independently in accordance with the inclusion criteria and collected the relevant demographic variables, clinical data, and laboratory findings at the time of admission. When the two physicians disagreed on the inclusion criteria, a third senior orthopedic surgeon intervened to determine and ultimately resolve the data inconsistencies.

(1) Demographic variables: age, sex, injured side
(2) Clinical data: pre-operative preparation time, comorbidity (none, one, two, and three or more), AO/OTA Fracture, and Dislocation Classification.
(3) Laboratory examination results: kalium (K), calcium (Ca), natrium (Na), balbumin (ALB), alkaline phosphatase (ALP), hemoglobin (HGB), total bilirubin (TBIL), creatinine (CREA), triglyceride (TG), high density lipoprotein cholesterol (HDLC), cystatin C (CYSC), white blood cell (WBC), platelet (PLT), and hypersensitive C-reactive protein (hsCRP). All results were obtained from the same laboratory of the Department of Laboratory Medicine of the Affiliated Hospital of Shandong University of Traditional Chinese Medicine. An identical test methodology was employed so as to achieve a consistent outcome.

We defined the waiting time for surgery of <48 h as early surgery and the waiting time for surgery of >48 h as delayed surgery (10).

### Statistical analysis

We utilized R software (version 4.4.0, IBM Corporation, Armonk, New York, United States) and SPSS (version 26, R Foundation for Statistical Computing, Vienna, Austria) for data analysis and statistical processing. Patient data were randomly divided into training and validation sets using the “caret” package in R. Descriptive statistics were generated through SPSS. Variables with a *p*-value of less than 0.05 in univariate analysis were included in the multivariate logistic regression. In the multivariate logistic regression analysis variables with a p-value of less than 0.05 were identified as independent predictors. These independent risk factors were then used to develop the nomogram model.

## Results

### Patient characteristics

A total of 1,116 patients with intertrochanteric fractures met the inclusion criteria to be included. The average age was 77.77 ± 11.19 years, with 762 (68%) being female and 354 (32%) male giving a male-to-female ratio of 1:2.15. Of the patients, 573 had left-sided fractures and 543 had right-sided fractures. The average waiting time for surgery was 5.46 ± 3.83 days. In the training set of 782 patients, 133 (17%) underwent early surgery, with 86 (65%) being female and 47 (35%) males. The remaining 649 patients (83%) underwent delayed surgery, comprising 458 (71%) females and 191 (29%) males. The validation set included 334 patients, of whom 56 (16%) received early surgery and 278 (84%) underwent delayed surgery. A summary of the characteristics of the variables in both the training and validation sets is provided in Table 1.

**Table 1:** Demographics of variables in the training and validation sets.

Variables	Training cohort (*N* = 782)	Validation cohort (*N* = 334)
Age		
18–40	11 (1.4%)	1 (0.3%)
40–65	89 (11.4%)	42 (12.6%)
65–75	157 (20.1%)	71 (21.3%)
75–85	326 (41.7%)	136 (40.7%)
≥85	199 (25.4%)	84 (25.1%)
Sex		
Male	238 (30.4%)	116 (34.7%)
Female	544 (69.6%)	218 (65.3%)
DVT		
Yes	188 (24%)	88 (24.3%)
No	594 (76%)	246 (73.7%)
Injured side		
Left	406 (51.9%)	168 (50.3%)
Right	376 (48.1%)	166 (49.7%)
Comorbidity		
0	91 (11.6%)	34 (10.2%)
1	87 (11.1%)	30 (9%)
2	119 (15.2%)	43 (12.9%)
≥3	485 (62%)	227 (68%)
Types		
A1	212 (27.1%)	97 (29%)
A2	532 (68%)	221 (66.2%)
A3	38 (4.9%)	16 (4.8%)
K(x̄ ± S)	3.89 ± 0.42	3.87 ± 0.41
Na(x̄ ± S)	138.10 ± 3.52	138.01 ± 3.50
Ca(x̄ ± S)	2.28 ± 3.60	2.15 ± 0.12
TBIL		
>22.4	229 (29.3%)	90 (26.9%)
≤22.4	553 (70.7%)	244 (73.1%)
CREA		
>73	174 (22.3%)	80 (24%)
≤73	608 (77.7%)	254 (76%)
TG		
>1.7	91 (11.6%)	39 (11.7%)
≤1.7	691 (88.4%)	295 (88.3%)
HDLC		
≥1.16	411 (52.6%)	180 (53.9%)
<1.16	371 (47.4%)	154 (46.1%)
CYSC		
>1.09	171 (21.9%)	73 (21.9%)
≤1.09	611 (78.1%)	261 (78.1%)
ALP		
>100	149 (19.1%)	75 (22.5%)
≤100	633 (80.9%)	259 (77.5%)
HGB		
0–30	3 (4%)	0 (0%)
30–60	3 (4%)	2 (6%)
60–90	141 (18%)	45 (13.5%)
90–120	423 (54.1%)	189 (56.6%)
≥120	212 (27.1%)	98 (29.3%)
hsCRP		
≥10	612 (78.3%)	251 (75.1%)
<10	170 (21.7%)	83 (24.9%)
ALB		
<40	397 (50.8%)	172 (51.5%)
≥40	385 (49.2%)	162 (48.5%)
PLT		
<100	28 (3.6%)	14 (4.2%)
100–300	678 (86.7%)	279 (84.5%)
>300	76 (9.7%)	41 (12.3%)
WBC		
>9.5	219 (28%)	93 (27.8%)
≤9.5	563 (72%)	241 (72.2%)

### Screening of predictors and nomogram development

**Figure 1:**
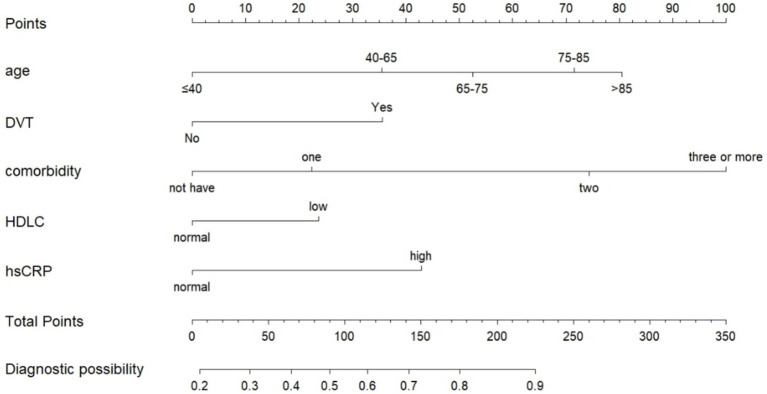
Nomogram for predicting prolonged preoperative waiting time of intertrochanteric fracture patients undergoing operative treatment.

We identified 20 factors associated with prolonged preoperative waiting time in patients with intertrochanteric fractures undergoing surgery, based on previous studies conducted by our research group (9), along with relevant literature and guidelines. These 20 variables from the training set were included in a univariate logistic regression analysis. Seven variables with *p*-value of less than 0.05 in the univariate logistic regression analysis were included in the multivariate logistic regression analysis. Using the step wise backward validation of multifactorial logistic regression analysis, a total of 5 factors with *p* < 0.05 were identified as independent risk factors when the Akaike Information Criterion was at its minimum (Table 2). The five selected variables age, deep vein thrombosis (DVT), comorbidity, high-density lipoprotein cholesterol (HDLC), and high-sensitivity C-reactive protein (hsCRP) were used to construct the predictive model. The expected outcome of the model is a patient preoperative wait time greater than 48 h. The high risk is that patients delay undergoing surgery. The low risk is that patients timely undergoing surgery. The model was then visualized as a nomogram using the “rms” package in R software (Figure 1).

**Table 2:** Univariate and multivariate logistic regression analysis of risk factors for prolonged preoperative waiting time of intertrochanteric fracture patients undergoing operative treatment.

	Univariate		Multivariate	
	OR (95% CI)	*P*	OR (95% CI)	*P*
Age	1.69 (1.41–2.03)	<0.001	1.20 (0.96–1.5)	0.048
Sex	0.76 (0.51–1.13)	0.178		
Injured side	0.96 (0.66–1.4)	0.841		
DVT	2.11 (1.26–3.54)	0.005	1.6 (0.93–2.75)	0.017
Comorbidity	1.95 (1.66–2.3)	<0.001	1.67 (1.38–2.04)	<0.001
Types				
A1		Refer		
A2	1.4 (0.95–2.07)	0.093		
A3	0.77 (0.33–1.76)	0.53		
K	1.02 (0.66–1.59)	0.929		
Ca	1.92 (0.44–8.27)	0.383		
Na	1.02 (0.97–1.07)	0.475		
TBIL	0.74 (0.5–1.1)	0.141		
CREA	0.98 (0.63–1.53)	0.926		
TG	1.04 (0.58–1.88)	0.887		
HDLC	1.86 (1.27–2.72)	0.001	1.66 (1.11–2.48)	0.014
CYSC	1.84 (1.09–3.08)	0.022		
ALP	0.96 (0.6–1.54)	0.873		
HGB	0.82 (0.63–1.07)	0.149		
hsCRP	2.34 (1.56–3.51)	<0.001	1.9 (1.23–2.94)	0.004
ALB	2.15 (1.46–3.18)	<0.001		
PLT	0.88 (0.53–1.47)	0.627		
WBC	1.82 (1.24–2.67)	0.710		

### Nomogram validation and clinical practice

**Figure 2:**
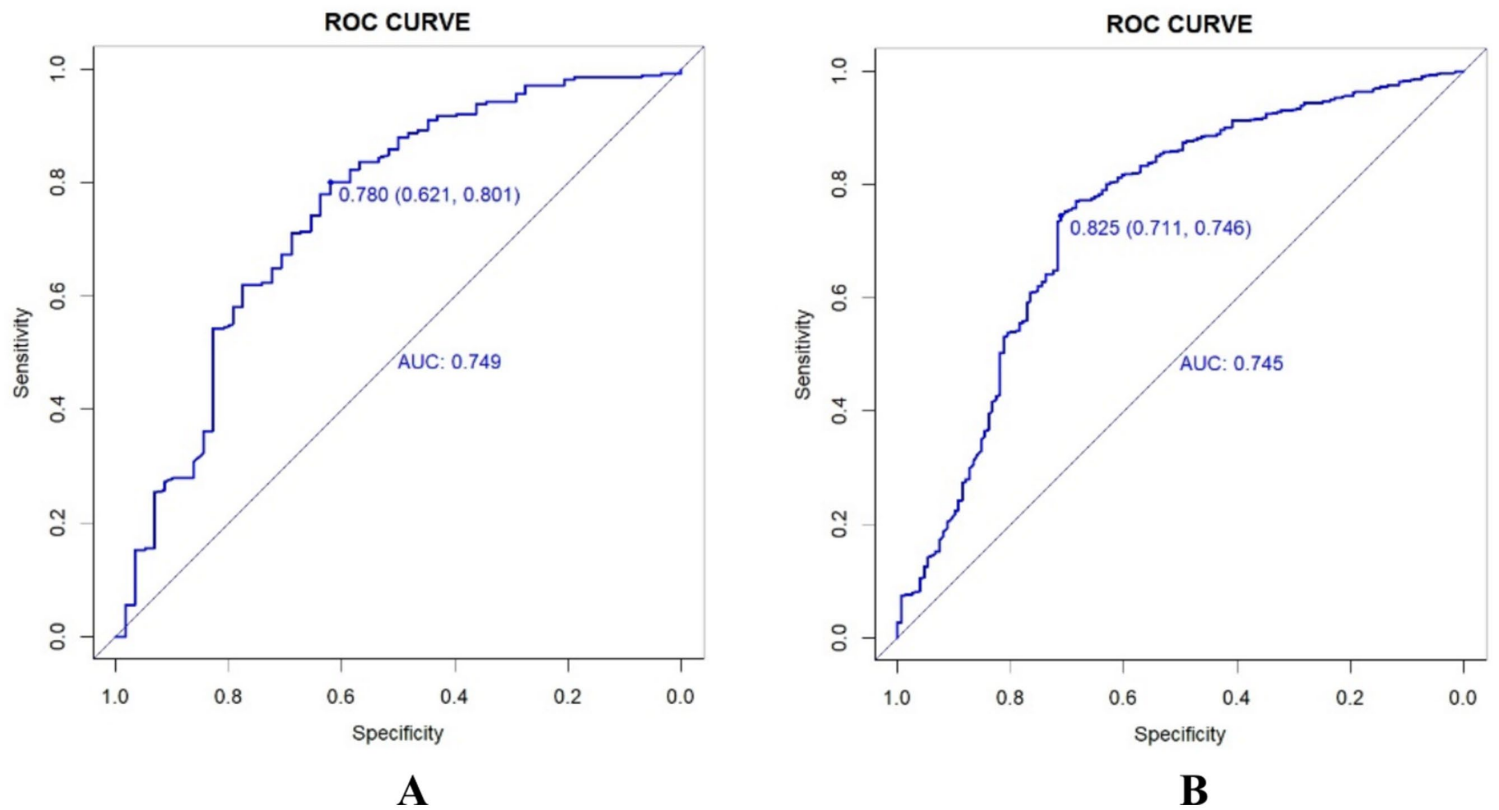
Receiver job characteristic curves for training sets **(A)** and validation sets **(B)**.

**Figure 3:**
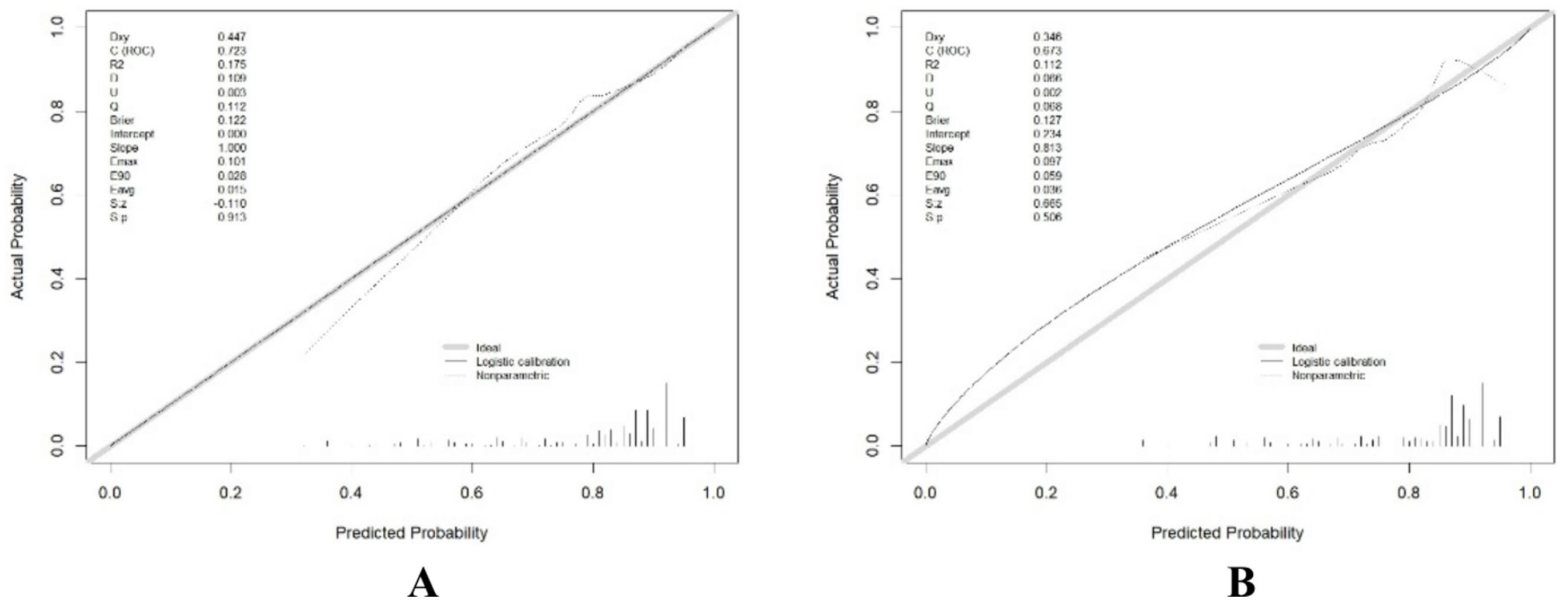
Comparison of calibration curves between the training set **(A)** and the testing set **(B)**.

**Figure 4:**
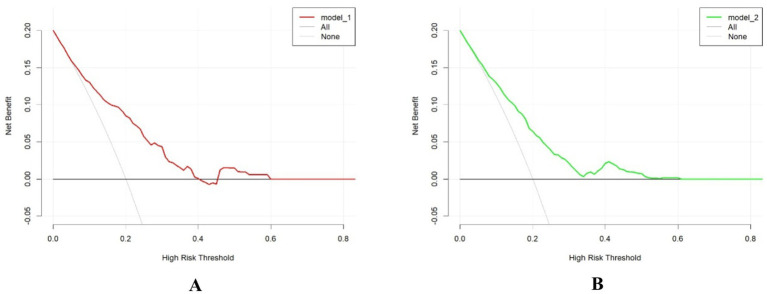
Comparison of decision curve analyses between the training set **(A)** and the testing set **(B)**.

Receiver operating characteristic curves, calibration curves, and decision curve analysis were generated for both the training and validation sets using the “pROC” and “rmda” packages, along with other methods in R software. After performing 500 bootstrap internal validations, the C-statistic for the training set was 0.799 and for the validation set, it was 0.727. The receiver operating characteristic curve is then plotted (Supplementary Figure S1). The receiver operating characteristic curves demonstrate that the model has excellent discriminatory ability (Figure 2). The calibration curves indicated strong agreement between the predicted and observed values for the preoperative waiting times in both the training and validation sets (Figure 3). The Hosmer–Lemeshow test yielded nonsignificant *p*-values of 0.729 and 0.211 for the two sets, respectively. The decision curve analysis curves revealed that the net benefit of using the nomogram to predict prolonged preoperative waiting times was maximized when threshold probabilities ranged from 0.02 to 0.60 for both sets. These results suggest that the model possesses strong clinical applicability and validity (Figure 4).

## Discussion

Our study identified age, DVT, comorbidities, HDL-C, and hsCRP as independent risk factors for prolonged preoperative waiting time in patients with intertrochanteric femur fractures. There was a positive correlation between delayed cases and high-risk factors, with more high-risk factors being more likely to delay undergoing surgery. These fractures are increasingly common among older adults and are associated with high mortality rates which impose significant burdens on patients, their families, and society. The 1-year postoperative mortality rate fractures is 15.6–23% (11, 12). Therefore, reducing preoperative waiting times could be critical intervention to lower mortality rates. Accurate prediction of surgical delays at admission may enable physicians to make more informed decisions regarding patient management. In our cohort, 189 patients (16.9%) underwent surgery within 48 h of admission, with an average waiting time of 5.6 days. This finding is consistent with that of the previous study by Li et al. (13). The mean age of the patients in our study was 77.77 years, with 66.8% being over 65 years old, and 25.4% being over 85 years. This age, distribution reflects an increasing in older adults, which is in line with the findings of previous studies (14, 15). Older age often complicates surgical procedures and reduces the body’s ability likely to tolerate surgery without intervention. The study found that 68.3% of patients were female, a proportion higher than that of patients. However, sex did not appear to influence preoperative waiting times. The number of left-sided and right-sided fractures was approximately equal, and our analysis indicated that the side of the injury did not significantly affect surgical delays. Nevertheless, some studies suggest that left-sided intertrochanteric fractures are associated with a higher risk of nonunion and reoperation, possibly due to the biomechanics of locking screws (16, 17). Further investigation is needed to determine if the side of the injury affects surgical duration and length of stay.

Venous thromboembolism is a serious complication of intertrochanteric fractures and a leading cause of mortality. The condition is primarily driven by venous stasis resulting from the fracture, endothelial injury, and systemic hypercoagulability due to trauma collectively known as the Virchow Triad. These factors contribute significantly to the development of DV (18). In our study, a quarter of the patients developed DVT preoperatively, a finding consistent with that of Song et al. (19) DVT affects 28.7% of individuals over the age of 65. Previous research has also identified several risk factors for DVT, including age, high-energy trauma, female sex, diabetes mellitus, chronic obstructive pulmonary disease, A3 typing, hidden blood loss, and abnormalities in lactate dehydrogenase, blood sodium, and hematocrit levels (20–22). To address DVT, prophylactic anticoagulants and inferior vena cava filters are commonly used. Anticoagulants are routinely administered to patients with intertrochanteric fractures at our hospital, yet some still develop DVT. Inferior vena cava filters, while effective, are costly and not affordable for all patients. Additionally, the prolonged course of anticoagulant therapy may contribute to extended preoperative waiting times, making patients more susceptible to DVT.

Our analysis indicates that comorbidities are significant risk factors for prolonged preoperative waiting times. Of the patients with three or more comorbidities, only 71 (10%) underwent surgery within 48 h. This delay may be attributed to the prevalence of comorbidities among older adults, many of which are respiratory and circulatory conditions that contribute to surgical postponements. Our study found that hypokalemia and hyponatremia at admission did not significantly affect preoperative waiting times. This is likely because these electrolyte imbalances can often be corrected quickly with medication. Conversely, HDL-C plays a critical role in regulating atherosclerosis through cholesterol reverse transport mechanisms and by influencing vascular endothelial function, inflammation, and innate immunity. Low HDL-C levels are associated with an increased risk of atherosclerotic cardiovascular disease (23, 24). We identified 11 patients with low HDL cholesterol who were diagnosed with atherosclerotic cardiovascular disease after admission. Low HDL-C levels have also been linked to a higher risk of DVT (25).

While our study did not specifically examine the impact of high HDL-C levels on preoperative waiting times, research by Lee et al. (26) suggests that elevated HDL-C levels reduce the likelihood of hip fractures. Additionally, elevated preoperative C-reactive protein (CRP) levels (>10 mg/dL) are associated with both short-term and long-term postoperative mortality in older adults with hip fractures (27, 28). High CRP levels are linked to an increased incidence of postoperative complications and higher mortality rates, making elevated CRP a plausible factor in prolonged preoperative waiting times for femoral neck fractures (29). At our hospital, hsCRP is routinely tested upon admission and is similar to CRP. Elevated hsCRP levels are associated with an increased risk of postoperative incision infections (30). Despite prophylactic antibiotic administration preoperatively, many doctors choose to delay surgery until hsCRP levels normalize.

Our analysis identified high levels of cystatin C as statistically significant (*p* < 0.05) in univariate regression analysis. Elevated cystatin C levels are associated with an increased risk of intertrochanteric fractures (31) and have been linked to postoperative sarcopenia, which leads to more complications in patients with hip fractures (32). Furthermore, high cystatin C levels are thought to be associated with increased mortality in older adults with hip fractures (33). Cystatin C reflects weakness and decreased physical function, potentially resulting in reduced surgical tolerance. Its lack of significance in multivariate regression analysis may be due to multifactorial interference or insufficient statistical power, indicating the need for further analysis with larger sample sizes. Hypoproteinemia was found to be statistically significant in univariate logistic regression analysis. It is associated with malnutrition and an increased number of comorbidities (34) and can also contribute to the development of DVT (35).

The predictive model we developed can help assess whether patients with intertrochanteric fractures in the future can receive timely surgical treatment, especially during physical examinations at community hospitals for older adults. Recording and filing data are crucial for effective geriatric hip management and care (36). Ensuring prompt hospital admission, particularly in rural areas for transfer to central medical facilities in cities, is essential (37). The model can be used alongside relevant measures to minimize preoperative waiting times. For high-risk patients identified upon arrival, implementing a fast-track care approach for patients with hip fractures can help shorten admission and surgery times (38). Additionally, a rapid reimbursement system is crucial for financially disadvantaged, high-risk patients (39). Based on previous studies, there is no need to reduce the use of warfarin analogs for patients at high risk of prolonged preoperative waiting times (40, 41). However, opioid use should be minimized in older adults who are at a higher risk of extended waiting periods (42). Multidisciplinary management, including the establishment of specialized geriatric orthopedic wards, can improve care for high-risk older adults (43). Reducing unnecessary auxiliary examinations, creating green channels for expedited care, and optimizing empirical drug use can help minimize delays due to comorbidities and poor underlying conditions. These strategies contribute to more effective surgical outcomes, shorter hospital stays, improved patient prognosis, and reduced economic burden.

## Limitations

The present study was a single-center retrospective analysis. This study focused only on patients with intertrochanteric femoral fractures in China, so limitations due to selection bias cannot be excluded. In addition, there is ongoing debate in the literature about the relevance of nomograms to different patient populations based on race (44). Our data is derived from real-world situations and may have imbalances that affect the effectiveness of the model. Due to the limited clinical information available, we could not analyze the impact of sociological factors such as patients’ lifestyle habits, educational level, and family members’ status on surgical delays. Moreover, we could not analyze emergencies that occurred during the treatment. Because the families of most of the deceased patients refused to communicate with us, the loss of follow-up made it impossible to analyze the mortality rate of the patients and the risk factors for death.

## Conclusion

Our study identified age, DVT, comorbidities, HDL-C, and ultrasensitive CRP as significant risk factors for prolonged preoperative waiting times in patients with intertrochanteric femur fractures. By developing a risk prediction model for prolonged preoperative waiting times, we can more accurately identify high-risk patients who may experience delays in surgery. This model enables the formulation of more personalized, scientific, and optimized treatment plans for these patients, ultimately aiming to improve outcomes and reduce delays.

## Data Availability

The original contributions presented in the study are included in the article/Supplementary material, further inquiries can be directed to the corresponding author.
