# Supplementary material for: Construction and validation of a predictive model for the risk of prolonged preoperative waiting time in patients with intertrochanteric fractures
Source: Front Med (Lausanne). 2025 Jan 17;11:1503719. doi: 10.3389/fmed.2024.1503719 (PMC11782220; doi:10.3389/fmed.2024.1503719)
Supplement: Supplementary file 1 [file Data_Sheet_1.docx]

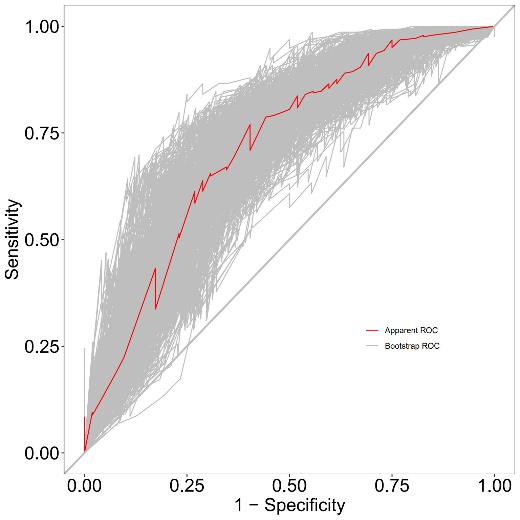

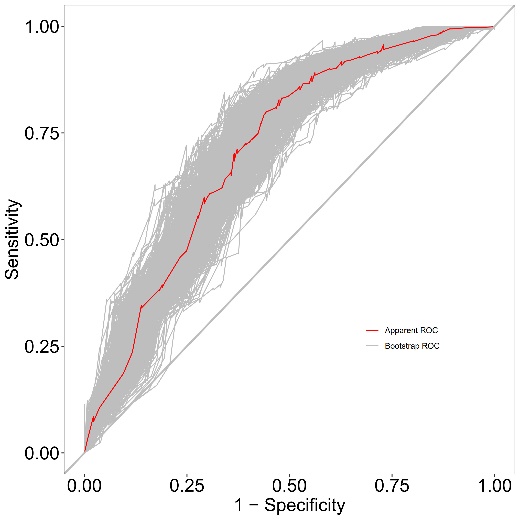


A B

**Fig. S1** After performing 500 bootstrap internal validations, receiver job characteristic curves for training sets (A) and validation sets (B).
